# Comparison of Intra-CL Injection and Peripheral Application of Prostaglandin F_2α_ Analog on Luteal Blood Flow and Secretory Function of the Bovine Corpus Luteum

**DOI:** 10.3389/fvets.2021.811809

**Published:** 2022-01-11

**Authors:** Agnieszka W. Jonczyk, Katarzyna K. Piotrowska-Tomala, Dariusz J. Skarzynski

**Affiliations:** Department of Reproductive Immunology and Pathology, Institute of Animal Reproduction and Food Research, Polish Academy of Sciences, Olsztyn, Poland

**Keywords:** prostaglandin F_2α_, corpus luteum, bovine, progesterone, oxytocin, blood flow, luteolysis

## Abstract

We investigated the effects of different doses of dinoprost injected directly into the bovine corpus luteum (CL) on (i) concentrations of progesterone (P_4_) and oxytocin (OT) in peripheral blood and (ii) mRNA levels of steroidogenic acute regulatory protein (STAR), cytochrome P450 family 11 subfamily A member 1 (P450scc), hydroxy-delta-5-steroid dehydrogenase, 3 β- and steroid delta-isomerase 1 (HSD3B), and receptor-interacting protein kinases 1 and 3 (RIPK1, RIPK3) in CL tissue. Moreover, we examined the effects of dinoprost, injected intra-CL or administered intramuscularly (IM), on CL secretory function and on indicators of CL vascular network status: luteal tissue area (LTA), CL blood flow (CLBF), and the CLBF:LTA ratio (Adj. CLBF), in cows at the early and mid-luteal phases. In the Experiment 1, cows (day 10 of the cycle) were allocated to (i) an intra-CL injection of saline (control; *n* = 3); (ii) an intra-CL injection of dinoprost (1.25 mg; 2.5 mg, or 5 mg; *n* = 3 for each dose); (iii) an IM administration of saline (control; *n* = 3); or (iv) an IM administration of dinoprost (25 mg; positive control; *n* = 3). Concentrations of OT and P_4_ were measured in plasma samples. The mRNA expression of steroidogenesis- or necroptosis-related factors was determined in CL tissue 4 h after treatments. In Experiment 2, cows on day 4 (*n* = 12) or day 10 (*n* = 12) were allocated to (i) an intra-CL injection of dinoprost (2.5 mg/0.5 ml; *n* = 6), or (ii) IM administration of dinoprost (25 mg/5 ml; *n* = 6). Concentrations of P_4_ were measured in plasma samples. Luteal tissue area, CLBF, and Adj. CLBF were assessed based on color Doppler ultrasonography. An intra-CL injection of dinoprost increased OT and decreased P_4_ levels in the jugular vein (JV) in a dose-dependent manner in cows at the mid-luteal phase. Increased CLBF and Adj. CLBF, accompanied by reduced P_4_ levels, were observed 2 h after intra-CL dinoprost injection in middle-stage CL. Decreased *STAR* and increased *RIPK1* and *RIPK3* mRNA levels confirmed that 2.5 mg dinoprost injected directly into CL is the minimum dose that induces luteolytic cascade. Injection of dinoprost directly into the CL (at a dosage lower than recommended for peripheral application) results in a pattern similar to IM dinoprost administration.

## Introduction

The corpus luteum (CL) plays the main role in regulation of the ovarian cycle and maintenance of pregnancy in mammalian species, because of progesterone (P_4_) production (1, 2). Many luteotrophic and luteolytic factors are involved in the life span of CL (1, 3–7). Development of the bovine CL is associated with intensive angiogenesis, which is crucial for its steroidogenic activity (8–10). Corpus luteum regression, on the other hand, consists of interruption of steroidogenesis (functional luteolysis) and demise of CL tissue due to cell death (structural luteolysis) (11, 12). Although apoptosis had been considered the most potent mechanism of cell death during luteolysis, a caspase-independent cell death pathway, necroptosis, was found recently to represent an alternative CL regression process in cows (13, 14).

Prostaglandin (PG) F_2α_ exerts stage-specific actions on bovine CL development, maintenance, and regression (15–18). During early luteal phase, PGF_2α_ may act as a luteo-supporting factor (18–20) and play a luteoprotective role (21–23). Uterine PGF_2α_ is necessary to induce luteolysis on days 17–18 of the estrous cycle in cattle, and initiates a new reproductive cycle (1, 24–27). Additionally, CL regression can be pharmacologically induced by the administration of exogenous PGF_2α_ in the middle stage of the estrous cycle, when CL is sensitive to PGF_2α_ (24, 28). During the early luteal phase, bovine CL is resistant to the luteolytic action of PGF_2α_ until day 5 of the estrous cycle (28–30).

Blood flow is a key regulatory component of CL function in cows (31). One method used to monitor luteal function during the estrous cycle of dairy cows is color Doppler sonography (32, 33). Changes in blood flow and P_4_ secretion in response to exogenous PGF_2α_ were previously examined in both early- and middle-stage CL (32, 34). On one hand, prior to the luteolytic cascade, PGF_2α_ increases luteal blood flow in cows (29). On the other hand, the lack of change in blood flow in the early-stage CL seems to be directly correlated with the resistance of CL to PGF_2α_ action during this period of the estrous cycle (31, 32).

The response of luteal steroidogenic cells may depend on local, direct effects of PGF_2α_ (autocrine/paracrine modes of action), or on indirect effects, including several regulatory mechanisms within the female reproductive tract (e.g., endocrine action, blood flow regulation, the contribution of the immune system, etc.) (35–37). Moreover, our previous study has demonstrated that intra-CL vs. intramuscular (IM) administration of PGF_2α_ differentially affects P_4_ secretion, modulating the main genes involved in angiogenic, steroidogenic, and cell death pathways in bovine CL (22, 23).

Prostaglandin F_2α_ analogs (e.g., dinoprost, cloprostenol, luprostiol, oestrophan) are well known as luteolytic drugs and are widely used in many synchronization protocols and manipulations of the estrous cycle in cows (38–41). Until now, IM has been the main route of administration used in veterinary practice. It was previously suggested that action of PGF_2α_ analogs on function of the middle-stage CL is dose-dependent (42). Therefore, in the present study, effects of different doses of dinoprost on bovine CL functions were examined at the mid-luteal phase. To the best of our knowledge, the effects of an intra-CL PGF_2α_ analog (dinoprost) on CL function as well as on luteal blood flow during the estrous cycle remain poorly understood. Furthermore, the intra-CL vs. peripheral actions of dinoprost on P_4_ output and local blood flow in bovine CL may depend on the phase of the estrous cycle. Therefore, the objective of the present study was to investigate the effects of different doses of dinoprost injected directly into bovine middle-stage CL on (i) concentrations of P_4_ and oxytocin (OT) in peripheral blood plasma samples, and (ii) mRNA levels of steroidogenic acute regulatory protein (STAR), cytochrome P450 family 11 subfamily A member 1 (P450scc), hydroxy-delta-5-steroid dehydrogenase, 3 β- and steroid delta-isomerase 1 (HSD3B) and receptor-interacting protein kinases 1 and 3 (RIPK1, RIPK3) in CL tissue. Moreover, we examined the effect of dinoprost either injected directly into CL or administered IM on CL secretory function and on indicators of the CL vascular network luteal tissue area (LTA), CL blood flow (CLBF), and the CLBF:LTA ratio (Adj. CLBF), in cows at the early (PGF_2α_-resistant) and mid-luteal (PGF_2α_-responsive) phases of the estrous cycle.

## Materials and Methods

### Animals and Surgical Procedures

For the present study, 42 healthy, cycling Polish Holstein-Friesian cows from a local commercial dairy farm (Smietki, Poland) were used. The herd calves all year and is monitored by trained veterinary and nutrition consultants. The history of the cows and the structure of the farms were investigated by a questionnaire for the owners. Written owner consent was obtained through the farm manager. The experiment was performed in a group of non-pregnant cows (650 ± 86 kg; 3–5 lactations; ages 5–7 years) that were considered for culling because of their low milk production. A tie-up system was use to contain the experimental cows. The animals were milked two times daily, and fed maize/grass-based full total mix ratio (TMR). Cows had *ad libitum* access to water and a salt-based mineral supplement. The absence of reproductive disorders was determined and general clinical examinations were performed in cows enrolled in the experiment. The estrous cycle was synchronized in all experimental cows by two IM injections of PGF_2α_ analog (dinoprost, 25 mg/5 ml; Dinolytic; Zoetis, Poland) over an 11-day interval, as reported previously (38). Follicular development and morphological changes of the CL during the estrous cycle were monitored with transrectal ultrasonographic visualization, and confirmed by observing visual signs of estrus (e.g., standing heat behavior and vaginal mucus discharge). The onset of estrus was taken as day 0 of the estrous cycle. Additionally, the stage of the estrous cycle was confirmed by radioimmunoassay (RIA) of P_4_ concentrations in blood plasma samples collected from the coccygeal vessels. The concentration of P_4_ in samples collected on day 0 of the estrous cycle was 0.41 ± 0.12 ng/ml (mean ± SEM).

### Polyvinyl Catheter Insertion

The animals were pre-medicated with xylazine (25–30 mg/animal; Xylavet 2%, ScanVet, Poland) followed by insertion of a polyvinyl catheter (outside diameter = 2.1 mm; inside diameter = 1.6 mm Tomel Sp, Poland) into the jugular vein (JV) to permit frequent blood sample collection, as described previously by Skarzynski et al. (43).

### Intra-CL Injection

Each cow was treated with 4 ml of 2% procaine hydrochloride (Polocainum Hydrochloricum; Biowet Drwalew, Poland) between the first and second coccygeal vertebrae for local epidural anesthesia. Intra-CL injections were administered under ultrasound guidance through a sterile needle 1.25 × 50 mm (18 G × 2″). The transducer and needle guide were coated with a sterile lubricant (Medicum, Poland) and positioned within the vagina. The ovary bearing the CL was positioned rectally to visualize it. The needle was then passed through the vaginal wall, and the injection of dinoprost or sterile saline was made directly into the CL. The intra-CL injection model was validated and described by Jonczyk et al. (22).

### Ovary Collection

Ovaries with CL were collected by colpotomy using a Hauptner's effeminator (Hauptner & Herberholz GmbH & Co. KG, Solingen, Germany), as described previously by Piotrowska et al. (44).

### Design of *in vivo* Experiments

#### Experiment 1

**Figure 1:**
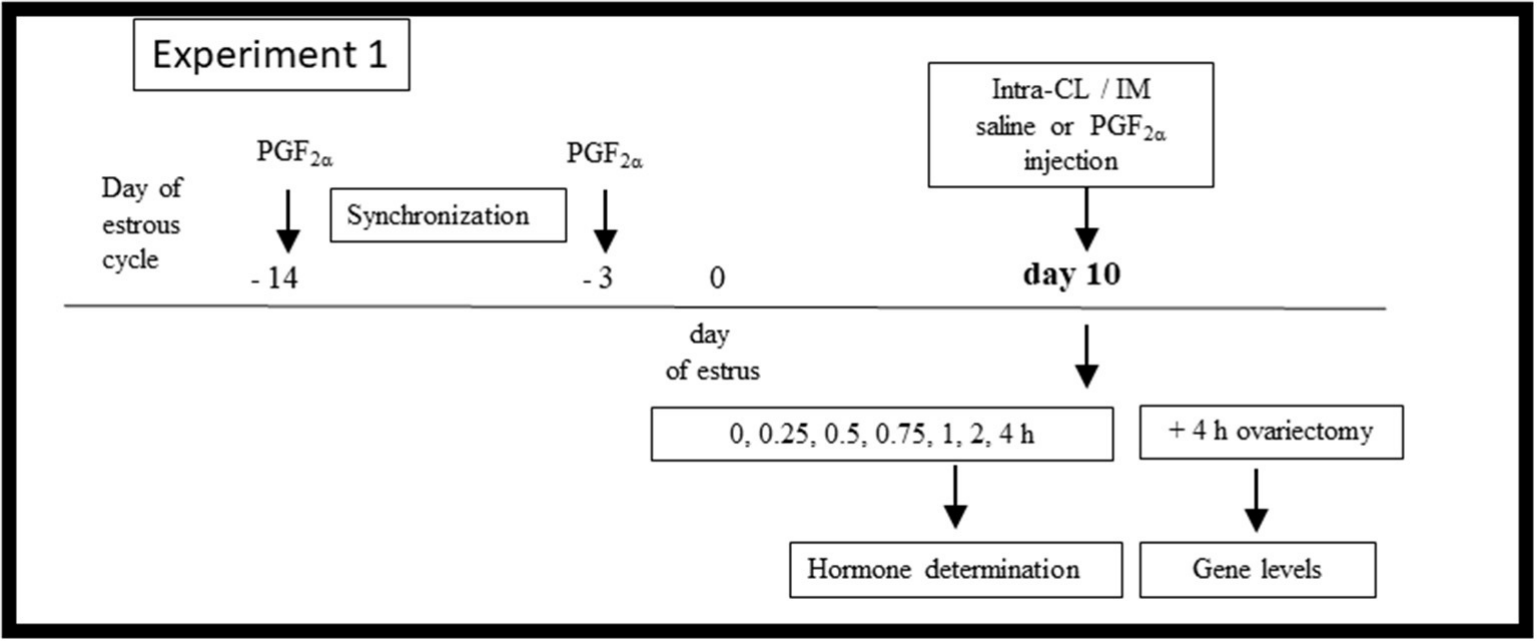
Schematic diagram of the Experiment 1 *in vivo* study design. Cows were synchronized via two injections of prostaglandin (PG) F_2α_ administered over an 11-day interval, starting on day −14 of the estrous cycle, with the onset of estrus considered day 0. On day 10 of the estrous cycle, the cows (*n* = 18) were allocated to (i) an intra-CL injection of sterile saline (control; *n* = 3), (ii) an intra-CL injection of three different doses of dinoprost (1.25 mg/0.5 ml, 2.5 mg/0.5 ml, and 5 mg/0.5 ml; *n* = 3 per dose), (iii) an IM administration of sterile saline (control; *n* = 3), (iv) an IM administration of dinoprost (25 mg/5 ml; positive control; *n* = 3). After treatment (time 0 h), blood plasma was collected over a 4-h period for hormone determination. At 4 h after treatment, the cows were ovariectomized. CLs were collected for evaluation of mRNA levels of specific genes.

Figure 1 shows the *in vivo* Experiment 1 study design. For frequent blood collection, a polyvinyl catheter was inserted into the JV of cows (*n* = 18) on day 9 of the estrous cycle, as described above. On day 10 of the estrous cycle, animals were randomly allocated to (i) an intra-CL injection of sterile saline (control; *n* = 3), (ii) an intra-CL injection of three different doses of dinoprost (1.25 mg/0.5 ml; 2.5 mg/0.5 ml, and 5 mg/0.5 ml; *n* = 3 per dose), (iii) an IM administration of sterile saline (control; *n* = 3), or (iv) an IM administration of dinoprost (25 mg/5 ml; positive control; *n* = 3). The time point of dinoprost or saline injection was defined as hour 0 of the experiment.

##### Dose-Dependent Effects of Intra-CL Injection of Dinoprost on Oxytocin and Progesterone Concentrations in the Jugular Vein of Cows in the Mid-Luteal Phase of the Estrous Cycle

Blood samples were collected from the JV of cows at 0, 0.25, 0.5, 0.75, 1, 2, and 4 h for determination of OT and P_4_ concentrations. Blood was collected into sterile 10-ml spray-coated K_2_EDTA tubes (BD Vacutainer, BD Diagnostic, Warsaw, Poland). After centrifugation (1,500 × *g*, 15 min at 4°C), the plasma was stored at −20°C until hormone concentrations were analyzed.

##### Dose-Dependent Effect of Intra-CL Injection of Dinoprost on mRNA Levels of Steroidogenesis- and Necroptosis-Related Factors in the Middle-Stage CL

The CL was collected by ovariectomy 4 h after treatments, to examine mRNA expression of steroidogenesis- or necroptosis-related factors in CL tissues. Each CL tissue was immediately placed into a 1.5 ml microcentrifuge tube containing 1 ml RNALater (#R0901, Sigma-Aldrich, Germany), homogenized, and stored at −80°C until quantitative real-time polymerase chain reaction (RT-qPCR) analysis was carried out.

#### Experiment 2

##### Effects of Intra-CL Injection of Dinoprost on Progesterone Concentration and Blood Flow in Cows at the Early and Mid-Luteal Phases of the Estrous Cycle, Compared to IM Dinoprost Administration

**Figure 2:**
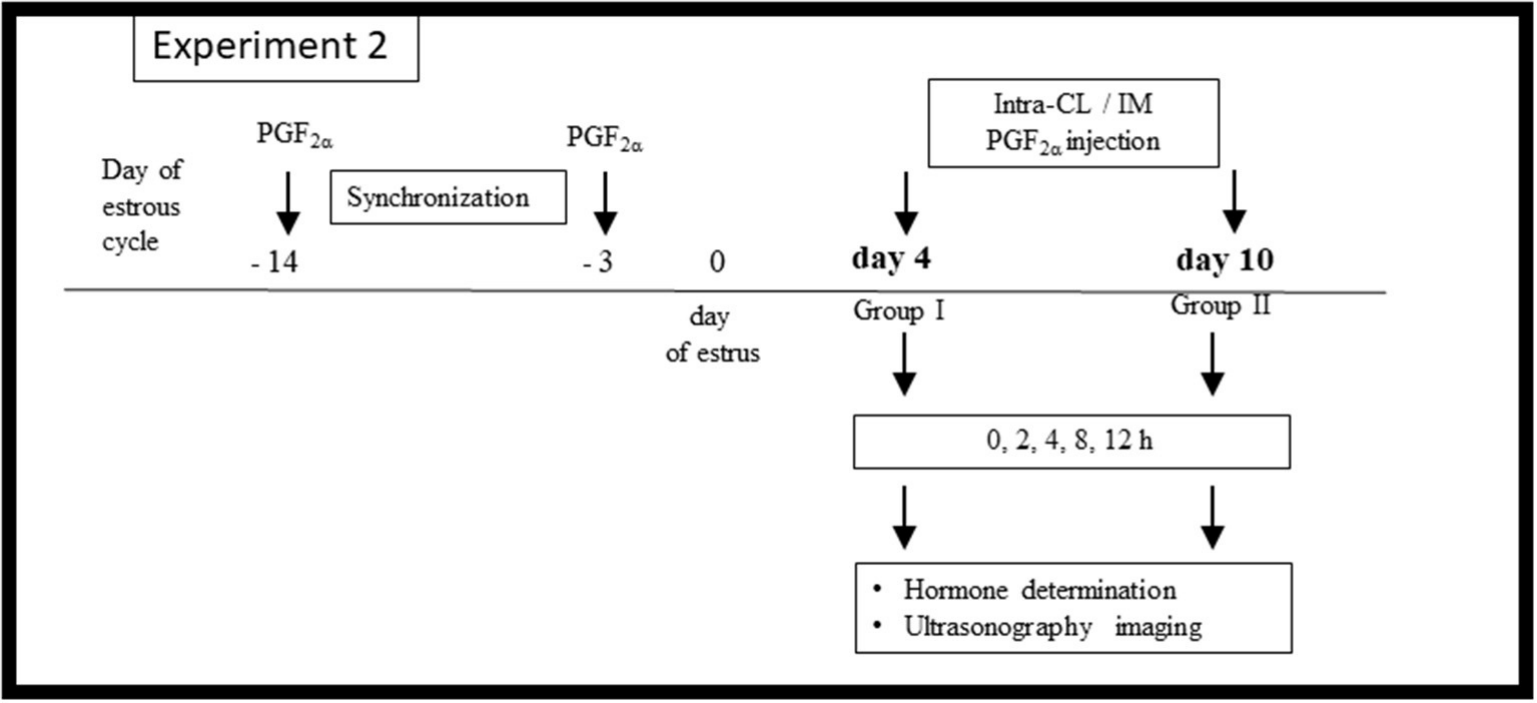
Schematic diagram of the Experiment 2 *in vivo* study design. Cows were synchronized via two injections of prostaglandin (PG) F_2α_ administered over an 11-day interval, starting on protocol day −14 of the estrous cycle, with the onset of estrus considered day 0. On day 4 (*n* = 12) or day 10 (*n* = 12) of the estrous cycle, the cows were allocated to (i) an intra-CL injection of dinoprost (2.5 mg/0.5 ml; *n* = 6 at each time point), (ii) IM administration of dinoprost (25 mg/5 ml; *n* = 6 at each time point). After treatment (time 0 h), blood plasma was collected over a 12-h period for hormone determination, and ultrasonographic imaging was performed.

Figure 2 shows the *in vivo* Experiment 2 study design. Cows (*n* = 24) were separated into two cohorts according to the phase of the estrous cycle: group I (early luteal phase; *n* = 12) and group II (mid-luteal phase; *n* = 12). A polyvinyl catheter was inserted into the JV for frequent blood collection on day 3 of the estrous cycle (group I) or on day 9 (group II), as described above. Afterwards, cows on day 4 (group I) or on day 10 (group II) were randomly allocated to (i) intra-CL injection of dinoprost (2.5 mg/0.5 ml; *n* = 6) or (ii) IM injection of dinoprost (25 mg/5 ml; *n* = 6).

Blood samples were collected from the JV 0, 2, 4, 8, and 12 h after dinoprost injection, for determination of P_4_ concentrations.

Bovine ovaries with active CL were examined 0, 2, 4, 8, and 12 h after dinoprost injection, by transrectal ultrasonography using an ultrasound scanner (MyLab 30VET Gold Color Doppler Diagnostic Ultrasound System, ESOATE Pie Medica, Genoa, Italy) equipped with a 7.5 MHz convex transducer. Ultrasonographic examination was carried out for blood flow mapping. Imaging settings (gain, depth, frequency, power, focus position, and frame rate) were standardized and remained unchanged for the duration of the experiment. Color-flow Doppler mapping modes were used to generate short video clips in which blood flow was detectable within the CL. Then videos were stored in the internal memory of the ultrasound machine for further computer-assisted image analyses.

### Hormone Determination

The OT concentrations in blood plasma were measured in duplicate via ELISA, as reported earlier by Skarzynski and Okuda (19), and using ice-chilled acetone for extraction (45). Non-commercial anti-OT serum was characterized previously (46), and used at a final dilution of 1:70,000. The standard curve ranged from 7.8 to 2,000 pg/ml. The intra- and inter-assay coefficients of variation (CV) were 9.5 and 11.3%, respectively.

The P_4_ concentration in blood plasma was measured in duplicate via direct RIA (P_4_^125^IRIA kit IM1188, Immunotech, Prague, Czech Republic) according to the manufacturer's instructions. The standard curve ranged from 0.1 to 100 ng/ml, and the effective dose for 50% inhibition (ED_50_) was 0.05 ng/ml. The intra- and inter-assay CVs were 6.2 and 8.8%, respectively.

### Computer-Assisted Image Analyses

Analyses of ultrasonographic images were adapted from Siqueira et al. (47). Briefly, LTA, CLBF, and Adj. CLBF were obtained from CL image characteristics. Areas of regions with CL or of the cavity (if present) were calculated, and the area of the cavity was subtracted from the CL area. For the quantitative assessment of vascularization of the CL, the area of colored pixels within the CL was determined, for indirect estimation of CLBF. An Adj. CLBF was calculated by dividing CLBF by LTA (ratio CLBF: LTA). All computer-assisted image analyses were performed using ZEN 2.6 (Carl-Zeiss, Germany), supported by ImageJ software.

### Quantitative Real-Time PCR

Total RNA was extracted from bovine CL tissues (40 ± 5 mg) using the Total RNA Mini Kit (#031-100, A&A Biotechnology, Gdansk, Poland) according to the manufacturer's instructions. The amount and purity of RNA were assessed using a NanoDrop 1000 (Thermo Fisher Scientific, ND-1000, Wilmington, DE, USA). The 260/280 nm absorbance ratio for all samples was approximately 2.0, and the 260/230 nm absorbance ratio ranged between 1.8 and 2.2. Then, 1 μg of each sample of total RNA was reverse-transcribed into cDNA using a QuantiTect Reverse Transcription Kit (#205311, Qiagen, Düsseldorf, Germany) according to the manufacturer's instructions.

Quantitative real-time PCR (RT-qPCR) assays were performed in an ABI 7900 HT sequence detection system using SYBR Green PCR master mix (Applied Biosystems, Foster City, CA, USA). The sequences for bovine glyceraldehyde-3-phosphate dehydrogenase (*GAPDH*), β-actin (*ACTB*), 18S ribosomal RNA (*RN18S1*), *STAR, P450scc, HSD3B, RIPK1*, and *RIPK3* were reported previously by Jonczyk et al. (22, 23). All primers were synthesized by Sigma-Aldrich (Custom Oligos Sigma-Aldrich, Haverhill, United Kingdom). Reference gene stability was determined using the NormFinder software program (MOMA, Aarhus University Hospital, Denmark), as previously described by Andersen et al. (48). Gene expression data are expressed relative to the best combination of two housekeeping genes (*ACTB/RN18S1*), and are presented as arbitrary units. The RT-qPCR results were analyzed using the method described by Zhao and Fernald (49). The total reaction volume was 10 μl, and contained 3 μl cDNA (10 ng), 1 μl each of forward and reverse primers (250 nM), and 5 μl SYBR Green PCR master mix (Applied Biosystems, Foster City, California, USA, #4309155). The RT-qPCR protocol was carried out as follows: initial denaturation (10 min at 95°C), followed by 45 cycles of denaturation (15 s at 95°C) and annealing (1 min at 60°C). After PCR amplification, melting curves were obtained by stepwise increases in temperature from 60 to 95°C, to ensure single product amplification. Control reactions without template or without primers were performed to confirm that products were free of primer dimers and genomic DNA contamination, respectively.

### Statistical Analysis

All experimental data are shown as means ± standard errors of the mean (SEM). The statistical analyses of the results of mRNA levels were performed using a non-parametric one-way ANOVA Kruskal-Wallis test followed by Dunnett's multiple comparison test (GraphPad Prism ver. 8.3.0; Graph Pad Software, San Diego, CA). The differences in P_4_ and OT concentrations in the venous blood plasma samples and analyses of indicators of vascular network status of the CL (LTA, CLBF, and Adj. CLBF) were analyzed using a repeated measures approach, in which treatments and time of sample collection (h) were treated as fixed effects, and all interactions were included (Supplementary Tables 1–3, two-way ANOVA test followed by the Sidak multiple comparison test; ANOVA; GraphPad Prism), as described previously (43).

## Results

### Experiment 1

#### Dose-Dependent Effects of Intra-CL Injection of Dinoprost on JV Oxytocin and Progesterone Concentrations in Cows in the Mid-Luteal Phase of the Estrous Cycle

Table 1 shows changes in OT concentrations in the peripheral blood plasma of cows after intra-CL injection of dinoprost at different doses, or IM administration (positive control) of dinoprost or saline solution, in the mid-luteal phase of the estrous cycle. Intra-CL injection of 1.25 mg dinoprost increased OT concentrations between 0.25 and 0.5 h after its injection, compared to the control group (*P* < 0.05; Table 1). After intra-CL injection of dinoprost at doses of 2.5 and 5 mg, increased OT concentrations were observed between 0.25 and 0.75 h after injection, compared to the control groups (*P* < 0.05; Table 1). In comparison, IM administration of 25 mg dinoprost enhanced OT concentrations between 0.25 and 0.75 h after treatment, compared to the control group (*P* < 0.05; Table 1).

**Table 1:** Effect of intra-CL injection or IM administration (positive control) of dinoprost or saline solution on oxytocin (OT) concentrations in blood plasma of cows (*n* = 3 per dose), on day 10 of the estrous cycle.

**Time** **(h)**	**Oxytocin (ng/ml)**					
	**Intra-CL injection**				**IM administration**	
	**Saline (control)**	**1.25 mg dinoprost**	**2.5 mg dinoprost**	**5 mg dinoprost**	**Saline (control)**	**25 mg dinoprost**
0	13.22 ± 0.1^a^	11.10 ± 3.74^a^	10.31 ± 1.74^a^	13.78 ± 3.28^a^	13.11 ± 0.06^a^	8.81 ± 1.00^a^
0.25	9.32 ± 4.25^a^	39.09 ± 14.25^b^	42.88 ± 8.08^b^^,^^*^	53.20 ± 12.77^b^^,^^**^	8.68 ± 4.16^a^	55.80 ± 14.55^b^^,^^***^
0.5	10.36 ± 2.59^a^	43.22 ± 9.85^b^^,^^*^	58.34 ± 7.68^b^^,^^***^	54.80 ± 18.93^b^^,^^**^	7.14 ± 0.69^a^	67.21 ± 15.15^b^^,^^***^
0.75	7.744 ± 0.27^a^	29.78 ± 7.98^a^	48.73 ± 1.98^b^^,^^**^	62.60 ± 19.36^b^^,^^***^	8.99 ± 1.27^a^	66.98 ± 13.97^b^^,^^***^
1	13.39 ± 2.42^a^	8.39 ± 3.77^a^	31.80 ± 7.20^a^	9.02 ± 3.04^a^	14.37 ± 1.45^a^	38.09 ± 2.45^a^^,^^*^
2	16.52 ± 4.15^a^	17.33 ± 3.23^a^	12.93 ± 8.26^a^	23.94 ± 2.80^a^	14.66 ± 4.54^a^	23.72 ± 6.41^a^
4	13.59 ± 1.61^a^	6.79 ± 4.39^a^	19.30 ± 6.37^a^	16.26 ± 4.97^a^	10.80 ± 3.23^a^	20.67 ± 9.50^a^

*All OT concentration values are expressed as mean ± SEM*.

*Letters*

(a, b) *indicate simple effects within rows (treated group compared to respective control group; P < 0.05)*.

*Asterisks*

(* *P < 0.05*;

** *P < 0.01*;

*** *P < 0.001) indicate statistically significant differences vs. the period before treatment (time 0 h), in two-way interactions*.

Table 2 shows changes in P_4_ concentrations in the peripheral blood plasma of cows after intra-CL injection at different doses, or IM administration (positive control) of dinoprost or saline solution, in the mid-luteal phase of the estrous cycle. Intra-CL injection of 2.5 mg dinoprost decreased P_4_ concentrations at 0.5 h and between 1 and 4 h after its injection, compared to the control group (*P* < 0.05; Table 2). Intra-CL injection of 5 mg dinoprost increased P_4_ concentrations at 0.25 h, then decreased its concentrations between 0.5 and 0.75 h and 2 and 4 h, respectively (*P* < 0.05; Table 2). An increase in P_4_ concentration was observed between 0.25 and 0.5 h, with a decline at 4 h after IM administration of dinoprost (*P* < 0.05; Table 2).

**Table 2:** Effect of intra-CL injection or IM administration (positive control) of dinoprost or saline solution on progesterone (P_4_) concentrations in blood plasma of cows (*n* = 3 per dose), on day 10 of the estrous cycle.

**Time** **(h)**	**Progesterone (ng/ml)**					
	**Intra-CL injection**				**IM administration**	
	**Saline**	**1.25 mg dinoprost**	**2.5 mg dinoprost**	**5 mg dinoprost**	**Saline**	**25 mg dinoprost**
0	9.53 ± 0.48^a^	9.77 ± 0.52^a^	9.37 ± 0.20^a^	8.92 ± 0.40^a^	8.65 ± 0.17^a^	9.39 ± 0.98^a^
0.25	9.13 ± 0.91^a^	9.22 ± 0.90^a^	8.19 ± 0.66^a^	11.99 ± 0.33^b^^,^^**^	8.53 ± 0.80^a^	11.83 ± 0.63^b^
0.5	8.89 ± 0.85^a^	8.78 ± 0.57^a^	7.98 ± 0.23^b^	7.23 ± 0.27^b^	8.55 ± 0.44^a^	11.42 ± 0.62^b^
0.75	9.13 ± 0.95^a^	7.16 ± 0.84^a^^,^^*^	7.35 ± 1.31^a^	6.36 ± 0.24^b^^,^^*^	8.73 ± 0.12^a^	9.72 ± 0.46^a^
1	8.49 ± 0.76^a^	6.87 ± 1.14^a^^,^^*^	5.12 ± 0.89^b^^,^^***^	6.19 ± 0.42^a^^,^^*^	8.56 ± 0.04^a^	7.83 ± 0.68^a^
2	9.04 ± 0.48^a^	8.99 ± 0.77^a^	4.35 ± 0.16^b^^,^^***^	6.46 ± 0.62^b^	8.29 ± 0.55^a^	6.19 ± 0.95^a^^,^^**^
4	8.51 ± 1.02	9.27 ± 0.90^a^	3.80 ± 0.71^b^^,^^***^	5.85 ± 0.33^b^^,^^*^	9.54 ± 0.29^a^	3.78 ± 0.77^b^^,^^***^

*All P_4_ concentration values are expressed as mean ± SEM*.

*Letters*

(a,b) *indicate simple effects within rows (treated group compared to respective control group; P < 0.05)*.

*Asterisks*

(* *P < 0.05*;

** *P < 0.01*;

*** *P < 0.001) indicate statistically significant differences vs. the period before treatment (time 0 h), in two-way interactions*.

#### Dose-Dependent Effects of Intra-CL Injection of Dinoprost on mRNA Levels of Steroidogenesis-and Necroptosis-Related Factors in the Middle-Stage CL

**Figure 3:**
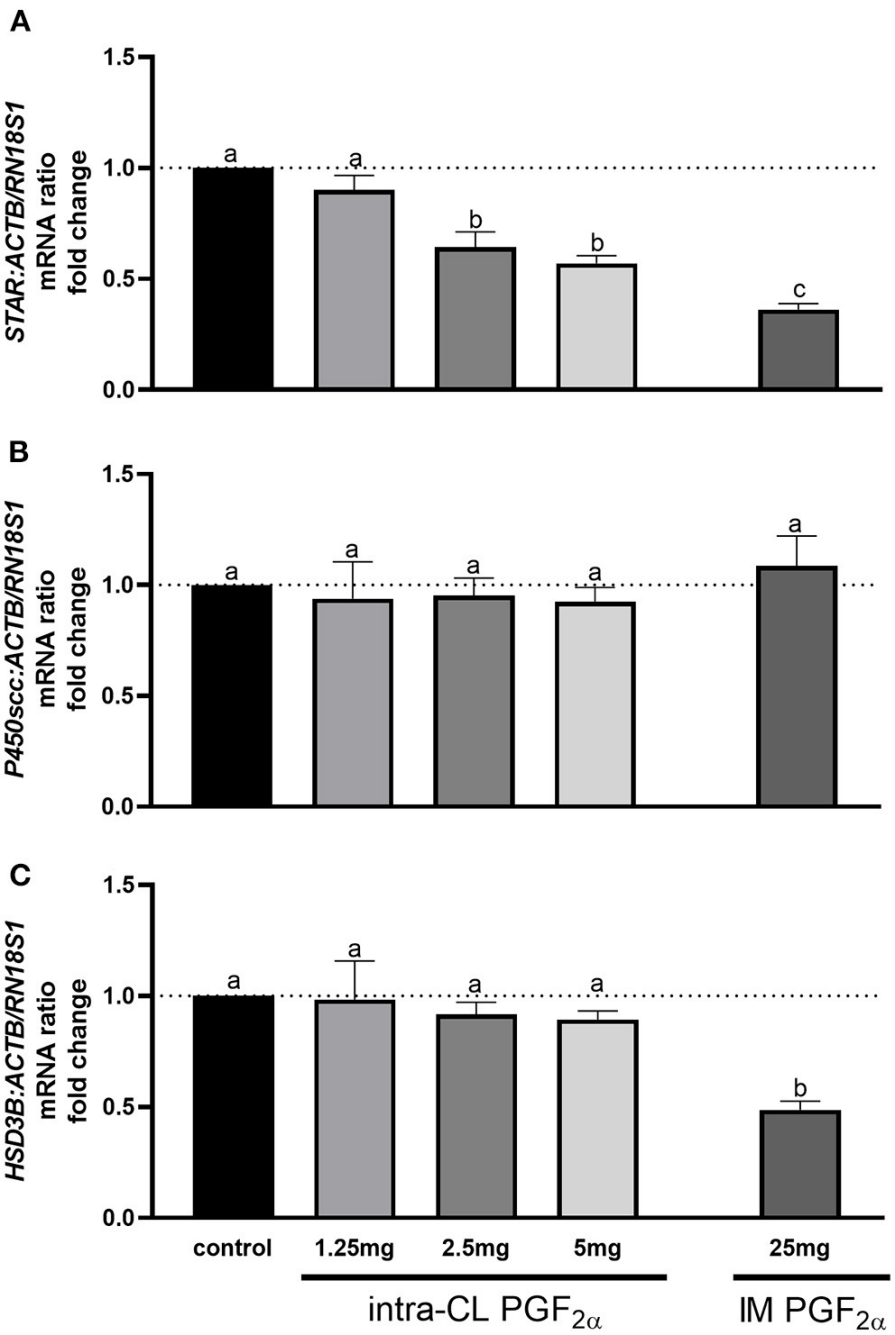
Effect of intra-CL injection or IM administration of dinoprost or saline solution on the mRNA levels of **(A)** steroidogenic acute regulatory protein (*STAR*), **(B)** cytochrome P450 family 11 subfamily A member 1 (*P450scc*), and **(C)** hydroxy-delta-5-steroid dehydrogenase, 3β- and steroid delta-isomerase 1 (*HSD3B*) in middle-stage corpora lutea (CL). Data are the mean ± SEM for three samples per treatment. Letters (^a−c^) indicate statistical differences between treatment groups (*P* < 0.05).

Figure 3 shows the results for quantitative analysis of mRNA levels of *STAR, P450scc*, and *HSD3B* in bovine middle-stage CL at 4 h after intra-CL injection of dinoprost at different doses, or IM administration (positive control) of dinoprost or saline solution. The intra-CL injection of dinoprost at doses of 2.5 and 5 mg downregulated *STAR* mRNA levels, compared to the control group (*P* < 0.05; Figure 3A). Furthermore, IM administration of dinoprost decreased *STAR* mRNA levels, compared to the control group (*P* < 0.05; Figure 3A). Additionally, lower *STAR* mRNA levels were observed after IM administration of dinoprost, compared to intra-CL injection of dinoprost at doses of 2.5 and 5 mg (*P* < 0.05; Figure 3A). Intramuscular administration of dinoprost downregulated *HSD3B* mRNA levels, compared to the control group, and also compared to all groups receiving dinoprost by intra-CL injection (*P* < 0.05; Figure 3C).

**Figure 4:**
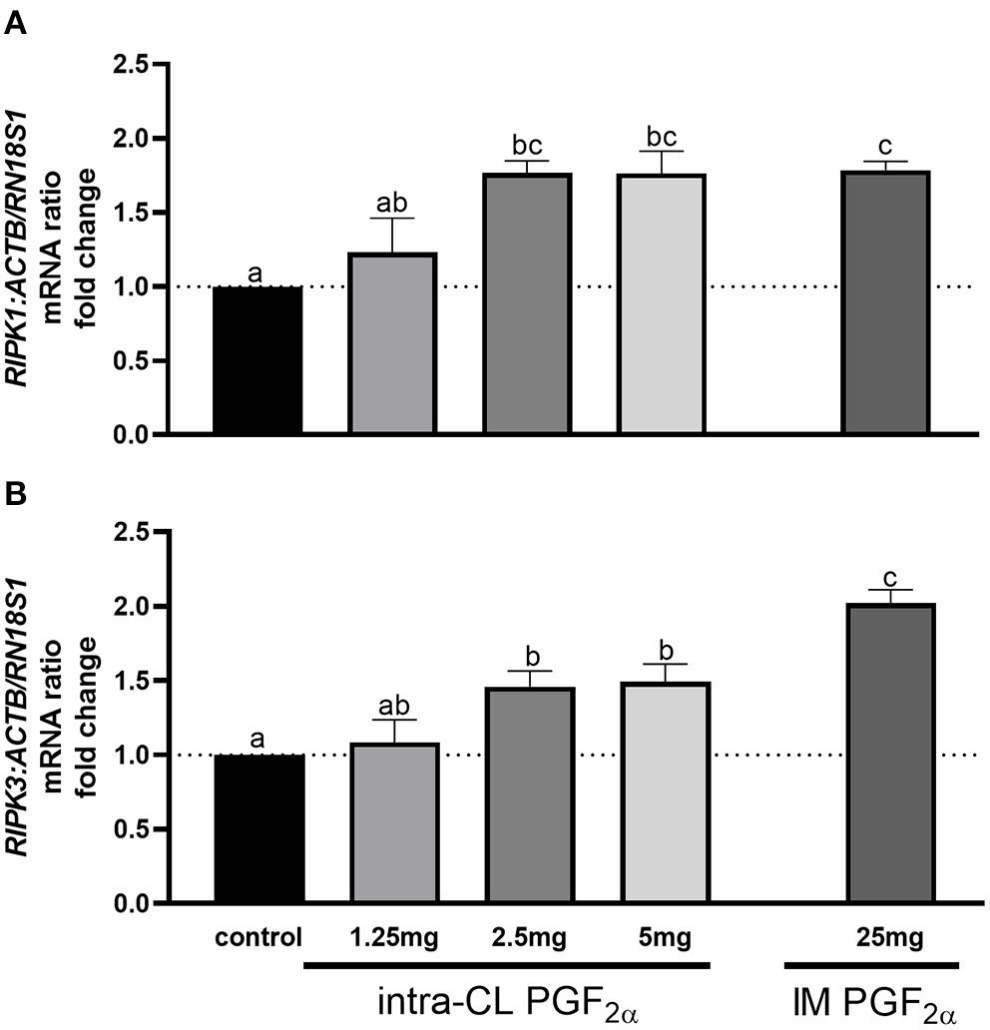
Effect of intra-CL injection of different doses of dinoprost on the mRNA levels of **(A)** receptor-interacting protein kinase 1 (*RIPK1*) and **(B)** receptor-interacting protein kinase 3 (*RIPK3*) in middle-stage corpora lutea (CL). Data are the mean ± SEM for three samples per treatment. Letters (^a−*c*^) indicate statistically significant differences between treatment groups (*P* < 0.05).

Figure 4 shows the results for quantitative analysis of mRNA levels of *RIPK1* and *RIPK3* in bovine middle-stage CL 4 h after intra-CL injection of dinoprost at different doses, or IM administration (positive control) of dinoprost or saline solution. The intra-CL injection of dinoprost at doses of 2.5 or 5 mg upregulated *RIPK1* and *RIPK3* mRNA, compared to the control group (*P* < 0.05; Figures 4A,B). Furthermore, *RIPK1* and *RIPK3* mRNA levels were enhanced after IM administration of dinoprost, compared to the control group (*P* < 0.05; Figures 4A,B). Additionally, greater *RIPK3* mRNA levels were observed after IM administration of dinoprost, compared to the mRNA levels after intra-CL injection of dinoprost at any of the doses tested (*P* < 0.05; Figure 4B). A dose of 2.5 mg dinoprost injected directly into CL was chosen as the luteolytic dose for further study.

### Experiment 2

#### Effects of Intra-CL Injection of Dinoprost on Progesterone Concentration and Blood Flow in Cows at the Early and Mid-Luteal Phases of the Estrous Cycle, Compared to IM Dinoprost Administration

**Figure 5:**
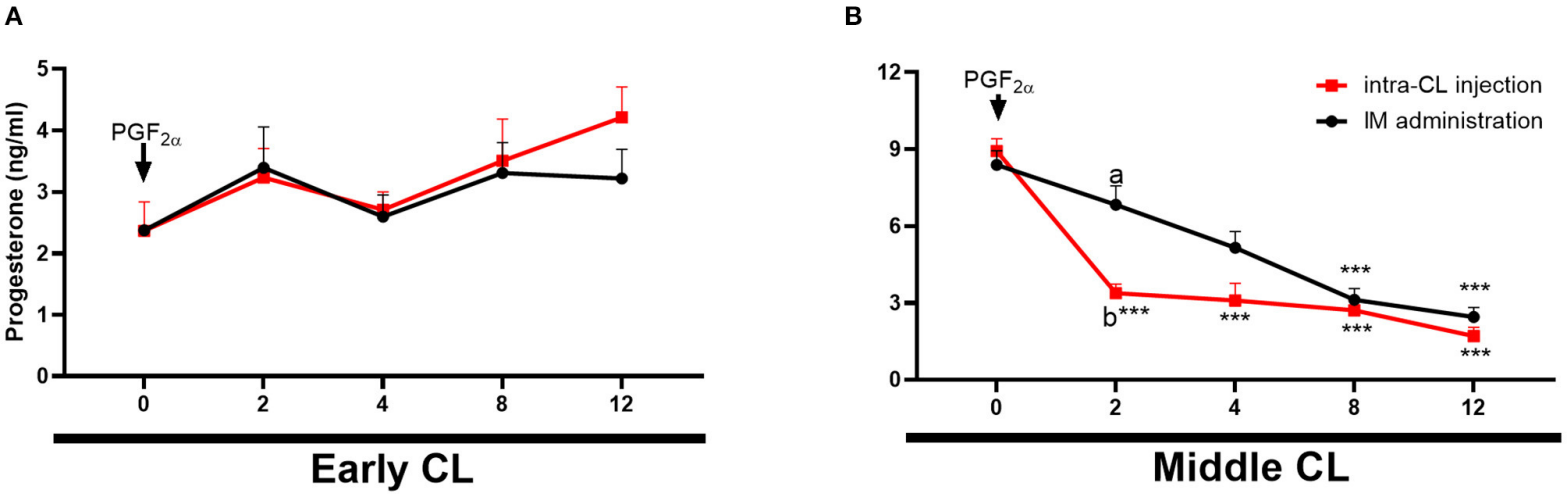
Effects of intra-CL injection (red lines) or IM administration (black lines) of dinoprost on progesterone (P_4_) concentrations in the blood in cows during the early **(A)** and middle **(B)** stages of the estrous cycle. Data are the mean ± SEM for six cows per treatment. Asterisks (***P < 0.001) indicate statistically significant differences vs. the period before treatment (time 0 h), in two-way interactions. Different subscript letters (^a,b^) indicate statistically significant differences (*P* < 0.05) between groups (intra-CL group vs. IM group) at the same time point, in three-way interactions.

Figure 5 shows changes in P_4_ concentrations in cows after intra-CL injection or IM administration of luteolytic doses of dinoprost. Blood plasma P_4_ concentrations decreased in cows at mid-luteal phase of the estrous cycle between 2 and 12 h after intra-CL injection of dinoprost, while its IM administration reduced P_4_ concentrations between 8 and 12 h, compared to the pre-treatment period (0 h) (*P* < 0.001; Figure 5B). Interestingly, P_4_ concentrations were lower 2 h after intra-CL injection of dinoprost than after the IM administration of dinoprost (*P* < 0.05; Figure 5B).

**Figure 6:**
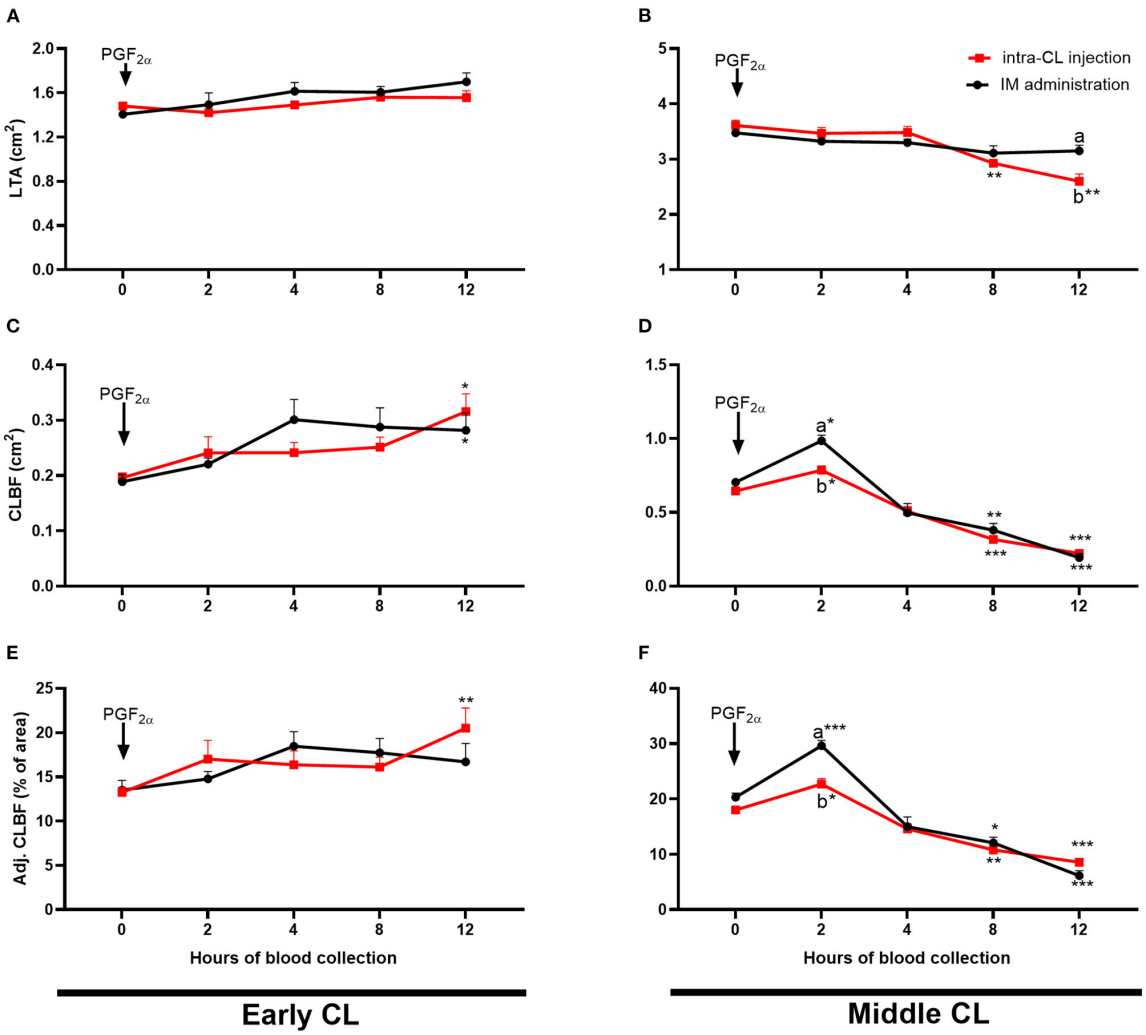
Effect of intra-CL injection (red lines) or IM administration (black lines) of dinoprost on luteal tissue area [LTA; **(A,B)**], amount of blood flow to the corpus luteum (CL) [CLBF; **(C,D)**], and CLBF:LTA ratio [Adj. CLBF in %; **(E,F)**] in cows during the early **(A,C,E)** and middle **(B,D,F)** stages of the estrous cycle. Data are the mean ± SEM for six cows per treatment. Asterisks (*P < 0.05; **P < 0.01; ***P < 0.001) indicate statistically significant differences vs. the period before treatment (time 0 h), in two-way interactions. Different subscript letters (^a,b^) indicate statistically significant differences (*P* < 0.05) between groups (intra-CL group vs. IM group) at the same time point, in three-way interactions.

Figure 6 shows temporal analysis of early- and middle-stage CL morphology and blood flow in cows after intra-CL injection or IM administration of luteolytic doses of dinoprost. In the early-stage CL, CLBF increased 12 h after either intra-CL injection of dinoprost or its IM administration, compared to the pre-treatment period (0 h) (*P* < 0.05; Figure 6C). Additionally, an increase in Adj. CLBF was observed 12 h after intra-CL injection of dinoprost, compared to the pre-treatment period (*P* < 0.05; Figure 6E). In the middle-stage CL, intra-CL injection of dinoprost reduced LTA between 8 and 12 h, compared to the pre-treatment period (0 h) (*P* < 0.01; Figure 6B). Both intra-CL injection of dinoprost and its IM administration increased CLBF and Adj. CLBF at 2 h, while both treatments decreased CLBF and Adj. CLBF between 8 and 12 h, compared to the pre-treatment period (0 h) (*P* < 0.05; Figures 6D,F). Interestingly, values of CLBF and Adj. CLBF were lower 2 h after intra-CL injection of dinoprost, compared to the IM administration of dinoprost (*P* < 0.05; Figures 5B,F,H). Moreover, LTA was lower 12 h after intra-CL injection of dinoprost vs. IM administration of dinoprost (*P* < 0.05; Figure 5D).

## Discussion

Regulation of the estrous cycle is widely applied in veterinary practice for estrus synchronization. The most effective routes and sites for administration of PGF_2α_ analogs, used for manipulation of the reproductive processes in breeding cows, have been the subject of numerous studies (40, 50, 51). Besides IM administration of PGF_2α_ analogs, their subcutaneous injection (SC) in the cervical area, or SC injection in the ischio-rectal fossa have also produced declines in P_4_ concentration, inducing luteolysis in the cow (40, 50, 51). Recently, we have noted that intra-CL injection of dinoprost reduced P_4_ levels at 4 h in the middle-stage CL, whereas it did not change P_4_ profiles in the early-stage CL (22). Moreover, peripheral application of PGF_2α_ analogs may be subject to side effects, and may have actions that differ from natural PGF_2α_ (38, 52–54).

The present study expands knowledge about luteolytic action of dinoprost applied directly into CL or IM, on PGF_2α_-resistant or PGF_2α_-responsive CL. It is well known that IM PGF_2α_ analogs induce the luteolytic process in cows. Therefore, our study used IM administration of dinoprost as a positive control. Interestingly, we showed that intra-CL injection of dinoprost affected not only secretory function of the bovine CL but also modulated luteal blood flow, depending on the phase of the estrous cycle. Moreover, to the best of our knowledge, we have provided the first evidence of dose-dependent effects of intra-CL injection of dinoprost on OT concentrations in JV blood plasma in the mid-luteal phase of the bovine estrous cycle.

It is well known that PGF_2α_ analogs stimulate rapid OT release from the CL in cattle (55, 56). Therefore, measurement of OT concentration in plasma after IM PGF_2α_ analog treatment is a good indicator of CL sensitivity to PGF_2α_ in cattle (42). Moreover, Skarzynski et al. (42) showed that the effect of IM administration of oestrophan on OT secretion was different on days 12 and 18 of the cycle, and it depended on the drug dosage. Therefore, to confirm the sensitivity of CL to intra-CL injection of different doses of dinoprost, OT concentrations were assessed in blood plasma in the middle-stage CL. Interestingly, in our study we observed that all doses of dinoprost injected directly into CL enhanced OT concentrations in blood plasma during the period of observation. Our results suggest that a rapid increase in OT concentration after intra-CL injection of dinoprost is the hallmark feature of the responsive CL. Therefore, we suspect that intra-CL injection is an adequate method to study the mechanism of PGF_2α_ actions on refractory and responsive bovine CL.

To determine luteolytic activity of different doses of dinoprost injected directly into CL, P_4_ concentrations were measured in blood plasma in cows at mid-luteal phase of the estrous cycle. Recently, Lopez-Gatius and Hunter (57) examined the action of different dinoprost doses (0.5 mg, 1.5 mg, and 2.5 mg) on CL function, reporting that intra-CL application of 2.5 mg dinoprost resulted in spontaneous twin reduction in pregnant cows by regression of CL. Moreover, Andrlikova et al. (58) studied effects of different doses of cloprostenol (5, 25, 50, and 100 μg) on the luteolytic process, showing that the minimal luteolytic dose of cloprostenol in cows, when injected directly into CL, is 10% of the recommended IM dose of the PGF_2α_ analog. In our study, 1.25 mg of dinoprost was ineffective when injected directly into middle-stage CL during 4 h of observation, while intra-CL injection of 2.5 or 5 mg of dinoprost induced luteolysis by reduction of P_4_ levels. Our above results suggest that 2.5 mg of dinoprost injected directly into the CL is a minimum luteolytic dose, which is 10% of the recommended IM dose of dinoprost.

To better understand the luteolytic action of PGF_2α_, we analyzed luteal blood flow and secretory function of the refractory or responsive CL after intra-CL injection of dinoprost, compared to the known actions of IM dinoprost. On one hand, in our study, IM administration of the luteolytic dose of dinoprost into middle-stage CL induced an increase, followed by a decrease, of indicators of the CL vascular network, evidence of certain early events of CL luteolysis in cows, a finding consistent with previous studies (32, 37). It had been previously shown that treatment with various doses of cloprostenol induced an acute increase in luteal blood flow 1 h after treatment, without dose-dependence, in the reduction of luteal volume in the mid-luteal phase (59). Our study is the first to compare the influence of intra-CL injection of dinoprost on blood flow in the early and mid-luteal phases of the estrous cycle. Interestingly, intra-CL injection of dinoprost showed parallel reductions in luteal blood flow and plasma concentrations of P_4_, relative to IM injection of a luteolytic dose of dinoprost. We should take into account that a drop in the level of P_4_ began 2 h after intra-CL injection of dinoprost, while the reduction in P_4_ concentration after dinoprost was administered IM occurred 8 h after treatment. Therefore, we assume that dinoprost injected directly into the CL may be more effective during PGF_2α_-induced luteolysis. We should highlighted that above results are relevant since a rapid decrease in the functionality of the CL is a better indicator of luteolysis than luteal tissue reduction. For instance, this may imply better results in synchronization protocols when PGF2α is used in combination with other hormones. The above results indicate that the method of intra-CL injection of PGF_2α_ analog may be successfully applied to breeding protocols in dairy cows. Our results are also in agreement with the previous report of Acosta et al. (32) showing that dinoprost administered IM was unable to cause CL regression when given in the early stage of the estrous cycle. Also, the study of Minela and Pursley (59) indicated that different doses of cloprostenol administered IM did not impair luteal development of the early-stage CL. Interestingly, in our study, increases in CLBF and Adj. CLBF were observed 12 h after intra-CL injection of dinoprost, compared to the pre-treatment period, in the early luteal phase of the estrous cycle. The above result confirmed that dinoprost did not induce luteolytic cascade, and did not disturb CL supply and its growth, when the drug was injected directly into early-stage CL. However, to clarify the effect of luteolytic PGF_2α_ on refractory CL, an additional *in vivo* study should be conducted that explores the mechanisms involved in early-stage CL secretory function in response to intra-CL injection of PGF_2α_ analog.

To better understand the molecular mechanisms underlying luteolysis, we examined the expression of genes involved in steroidogenesis and cell death (necroptosis) in response to different doses of dinoprost injected directly into the CL. It is important to know that acute changes in steroidogenesis appear to be primarily associated with changes in active STAR protein, without changes in activities of other steroidogenic enzymes (60). Moreover, Shirasuna et al. (15) demonstrated that IM administration of PGF_2α_ analog decreased *STAR* mRNA transcription 0.5 h after treatment in the center and periphery of the middle-stage CL. Recently, we have shown that dinoprost, injected directly into CL or administered IM, downregulated *STAR* mRNA levels 4 h after treatment in the middle-stage CL (22). Importantly, our study found that intra-CL injection of 1.25 mg dinoprost was not effective in reducing the steroidogenesis pathway, while doses of 2.5 and 5 mg of dinoprost decreased *STAR* mRNA levels. Thus, early stages of the steroidogenic pathway were blocked. The latest investigations have demonstrated necroptosis (in addition to apoptosis) as a mechanism of the cell death during CL regression (13, 14, 23). Hojo et al. (13) showed that RIPK1 and RIPK3 (the main genes playing roles in necroptotic processes) are strongly elevated in the bovine CL during both spontaneous and IM PGF_2α_-induced luteolysis. Our previous study reported that dinoprost, injected directly into CL or administered IM, enhanced indicators of necroptosis and apoptosis 4 h after the treatment in the middle-stage CL (23). Interestingly, in our present study, we observed dose-dependent effects of intra-CL injection of dinoprost on genes involved in necroptosis, i.e., intra-CL injection of 1.25 mg dinoprost did not affect the necroptosis pathway, while 2.5 and 5 mg doses of dinoprost up-regulated the relative mRNA levels of *RIPK1* and *RIPK3*. Analyses of expression of genes involved in steroidogenesis or necroptosis showed that the smallest dose of dinoprost injected directly into CL was not related to their effects. Additionally, 2.5 mg of dinoprost injected into the CL was confirmed as the minimum effective dose to induce CL regression, and may be used in veterinary practice to manipulate the estrous cycle.

The method of intra-CL application of drugs/hormones could be a prevalent tool in bovine reproduction. It is important to know that the dairy and beef cow industry economies depend on fertility, and this has been the subject of numerous studies (40, 50, 51, 61). Quality assurance of beef in particular seeks to minimize meat damage to preserve its value, by minimizing the use of hormones in beef production (40). The previous studies of Chebel et al. (50) and Mezera et al. (51), which focused on differences between SC and IM administration, indicated no differences in cows that underwent complete luteolysis. These authors suggested that the SC method of dinoprost administration is acceptable for CL regression. Our results highlight that injection of dinoprost directly into the CL (at lower dose than would be recommended for IM administration) results in a similar physiological response to that produced by IM dinoprost administration. Therefore, we suspect that administration of PGF_2α_ analogs directly into the CL to induce luteolysis may be incorporated into synchronization protocols. This method might have a huge economic value in the dairy and beef cow industries. However, further studies are still needed to understand the influence of intra-CL injection of dinoprost on the duration of the estrous cycle.

In conclusion, the present study demonstrated that intra-CL injection of dinoprost increases OT concentrations and decreases JV P_4_ levels, in a dose-dependent manner, in cows at the mid-luteal phase of the estrous cycle. An increase in indicators of vascularization of CL (CLBF and Adj. CLBF), accompanied by a drop in P_4_ level, was observed 2 h after intra-CL dinoprost injection in the middle-stage CL. Moreover, the lack of changes in blood flow and P_4_ concentration at the early luteal phase of the estrous cycle appeared to be directly correlated with the resistance of CL to the action of dinoprost injected directly into the early-stage CL. Furthermore, the decrease in *STAR* mRNA and increases in *RIPK1* and *RIPK3* mRNA levels confirmed that 2.5 mg of dinoprost injected directly into CL is a minimum dose that will induce the luteolytic cascade. We expect that our results could provide new knowledge to optimize breeding protocols in cows.

## Data Availability Statement

The original contributions presented in the study are included in the article/Supplementary Materials, further inquiries can be directed to the corresponding author/s.

## Ethics Statement

The animal study was reviewed and approved by University of Warmia and Mazury in Olsztyn, Poland (Agreement No. 23/2012/N). Written informed consent was obtained from the owners for the participation of their animals in this study.

## Author Contributions

AWJ, KKP-T, and DJS: investigation and writing—review and editing. AWJ and KKP-T: methodology, formal analysis, data curation, visualization, and writing—original draft. DJS: conceptualization, supervision, and funding acquisition. All authors have read, critically revised, and approved the final version of the manuscript.

## Funding

The research was supported by a grant from the National Science Center in Poland (DEC-2011/03/B/NZ9/01634).

## Conflict of Interest

The authors declare that the research was conducted in the absence of any commercial or financial relationships that could be construed as a potential conflict of interest.

## Publisher's Note

All claims expressed in this article are solely those of the authors and do not necessarily represent those of their affiliated organizations, or those of the publisher, the editors and the reviewers. Any product that may be evaluated in this article, or claim that may be made by its manufacturer, is not guaranteed or endorsed by the publisher.

## Abbreviations

CL: corpus luteum
P_4_: progesterone
PGF_2α_: prostaglandin F_2α_
IM: intramuscular
OT: oxytocin
LTA: luteal tissue area
CLBF: corpus luteum blood blow
Adj. CLBF: adjusted corpus luteum blood flow
RIA: radioimmunoassay
JV: jugular vein
RT-qPCR: quantitative real-time polymerase chain reaction
*GAPDH*: glyceraldehyde-3-phosphate dehydrogenase
*ACTB*: β-actin
*RN18S1*: 18S ribosomal RNA
*STAR*: steroidogenic acute regulatory protein
*P450scc*: cytochrome P450 family 11 subfamily A member
*HSD3B*: hydroxy-delta-5-steroid dehydrogenase, 3 β- and steroid delta-isomerase 1
*RIPK1*: receptor-interacting protein kinases 1
*RIPK3*: receptor-interacting protein kinases 3
ELISA: enzyme-linked immunosorbent assay
SC: subcutaneous injection.
