# Supplementary material for: Comparison of Intra-CL Injection and Peripheral Application of Prostaglandin F2α Analog on Luteal Blood Flow and Secretory Function of the Bovine Corpus Luteum
Source: Front Vet Sci. 2022 Jan 11;8:811809. doi: 10.3389/fvets.2021.811809 (PMC8787071; doi:10.3389/fvets.2021.811809)
Supplement: Supplementary file 1 [file Data_Sheet_1.docx]

Supplementary Material

# Supplementary Tables

|  | **Treatments** | **Time** | **Interaction** |
| --- | --- | --- | --- |
| Progesterone | 12.50 ^***^ | 17.81 ^***^ | 4.903 ^***^ |
| Oxytocin | 16.65 ^***^ | 14.02 ^***^ | 2.682 ^***^ |

**Supplementary Table 1.** Two-way ANOVA *F* value in the middle stage CL: Treatments ( intra-CL saline, 1.25 mg dinoprost; 2.5 mg dinoprost, 5 mg dinoprost, IM saline, 25 mg dinoprost) *vs* Time (0, 0.25, 0.5, 0.75, 1, 2, 4 h). *DF*: treatments, 5; time, 6; interaction, 30, ^***^ p < 0,001.

|  | **Treatments** | **Time** | **Interaction** |
| --- | --- | --- | --- |
| Progesterone | 0.3439 | 3.287 ^*^ | 0.5295 |
| Luteal tissue area | 1.086 | 6.412 ^**^ | 1.835 |
| CL blood flow (cm^2^) | 0.1172 | 6.207 ^**^ | 1.424 |
| Adj. CL blood flow (% of area) | 0.07823 | 4.253 ^**^ | 1.772 |

**Supplementary Table 2.** Two-way ANOVA *F* value in the early stage CL: Treatments ( intra-CL, IM) *vs* Time (0, 2, 4, 8, 12 h). *DF*: treatments, 1; time, 4; interaction, 4, ^*^ p < 0,05; ^**^ p < 0,01.

|  | **Treatments** | **Time** | **Interaction** |
| --- | --- | --- | --- |
| Progesterone | 5.252 ^*^ | 89.25 ^***^ | 8.224 ^***^ |
| Luteal tissue area | 0.7946 | 17.72 ^***^ | 5.409 ^**^ |
| CL blood flow (cm^2^) | 5.225 ^*^ | 130.1 ^***^ | 3.722 ^*^ |
| Adj. CL blood flow (% of area) | 6.492 ^*^ | 117.2 ^***^ | 6.492 ^***^ |

**Supplementary Table 3.** Two-way ANOVA *F* value in the middle stage CL: Treatments ( intra-CL, IM) *vs* Time (0, 2, 4, 8, 12 h). *DF*: treatments, 1; time, 4; interaction, 4, ^*^ p < 0,05; ^**^ p < 0,01; ^***^ p<0.001.
